# Plastic deformation mechanisms in a severely deformed Fe-Ni-Al-C alloy with superior tensile properties

**DOI:** 10.1038/s41598-017-15905-5

**Published:** 2017-11-15

**Authors:** Yan Ma, Muxin Yang, Ping Jiang, Fuping Yuan, Xiaolei Wu

**Affiliations:** 1 State Key Laboratory of Nonlinear Mechanics, Institute of Mechanics, Chinese Academy of Sciences, Beijing, 100190 China; 2 School of Engineering Science, University of Chinese Academy of Sciences, Beijing, 100049 China

## Abstract

Nanostructured metals have high strength while they usually exhibit limited uniform elongation. While, a yield strength of approximately 2.1 GPa and a uniform elongation of about 26% were achieved in a severely deformed Fe-24.8%Ni-6.0%Al-0.38%C alloy in the present work. The plastic deformation mechanisms for the coarse-grained (CG) sample and the cold-rolled (CR) samples of this alloy were investigated by a series of mechanical tests and microstructure characterizations before and after tensile tests. No obvious phase transformation was observed during the tensile deformation for the CG sample, and the plastic deformation was found to be mainly accommodated by deformation twins and dislocation behaviors. While significant phase transformation occurs for the CR samples due to the facts that the deformed grains by CR are insufficient to sustain the tensile deformation themselves and the flow stress for the CR samples is high enough to activate the martensite transformation. The amount of phase transformation increases with increasing thickness reduction of CR, resulting in excellent tensile ductility in the severely deformed alloy. The back stress hardening was found to play a more important role in the CR samples than in the CG sample due to the dynamically reinforced heterogeneous microstructure by phase transformation.

## Introduction

Grain refinement has been extensively utilized to strengthen metals and alloys^1–5^. Bulk ultrafine-grained (UFG) and nanostructured (NS) metals can be many times stronger when compared to their conventional coarse-grained (CG) counterparts^1–5^, but with poor strain hardening and limited ductility. Previous studies have shown that several strategies can be employed to obtain both high strength and good ductility in metals and alloys, such as gradient structures^6–8^, heterogeneous lamella structure^9,10^, nanotwins with coherent twin boundaries (CTB)^11,12^, nano-precipitates^13,14^, bimodal/multimodal structure^15,16^, twinning-induced plasticity (TWIP)^17,18^, transformation-induced plasticity (TRIP)^19,20^, and lattice softening by composition control^21–25^. Recently, a new design to lattice-softened alloys with both ultrahigh strength and high ductility has been proposed^26,27^, in which C is added as stabilizer for the austentite phase, Al is added as stabilizer for the bcc phase and the final chemical composition is Fe-24.8%Ni-6.0%Al-0.38%C (in wt.%). The high strength has been attributed to the grain refinement by severe plastic deformation (SPD), the high strain hardening and the high ductility have been attributed to the appropriate choice of chemical composition for the lattice softening and the multimodal-structure formation^27^.

The mechanism of strain hardening for most ultrahigh strength steels is still not fully clear since their plastic deformation is inhomogeneous due to the heterogeneous microstructure^28,29^. Even for an alloy with initial single austentite phase and homogeneous grain size, martensite transformation or deformation twins would make the plastic deformation inhomogeneous and the strain hardening behavior complex^17,19^. In our recent work^10^, the high strain hardening and the high ductility in a commercial pure Ti with heterogeneous lamella structure were attributed to the back-stress hardening associated with the plastic incompatibility between the lamellae with different grain sizes. In our more recent paper^30^, the extraordinary strain hardening rate in a dual-phase high specific strength steel (HSSS) can be attributed to the high back stresses that arise from load transfer and strain partitioning between the two phases with different mechanical properties. The back stress hardening due to the high internal stresses have in fact been reported in TWIP steels^18,31,32^, TRIP steels^33^, and dual-phase alloys^30,34,35^ to account for the strong strain hardening and the large ductility.

Indeed, the plastic deformation in the Fe-24.8Ni-6.0Al-0.38 C (in wt.%) alloy with heterogeneous microstructure should be similar to that in composites, and can be characterized by the strong back stress hardening due to the load redistribution and the strain partitioning between the constituent phases. In this regard, we analyze the mechanisms of plastic deformation and the strain hardening due to the back stress in the Fe-24.8Ni-6.0Al-0.38 C (in wt.%) alloy consisting of *γ* austentite phase and *α*′ martensite phase in this paper. The initial CG materials were prepared by melting under Ar atmosphere and solution treatment at 1373 K for 24 h following quenched in water. Then different heterogeneous microstructures with varying yield strength were introduced by cold rolling (CR) with different thickness reduction. A series of load-unload-reload (LUR) tests have been conducted to investigate the back stress evolutions during the tensile deformation for various microstructures, and then the mechanisms of back stress hardening for various microstructures have been carefully analyzed. Generally, martensite transformation can be considered as a stress-induced process based on the thermodynamic action with a local threshold stress during the transformation^17,36^. The transformation behaviors should be different for various microstructures with different flow stresses, then the quantitative analysis for the martensite transformation of various microstructures has been conducted by a series of X-ray diffraction (XRD) measurements before and after tensile tests. The detailed microstructure evolutions have also been obtained by electron backscattered diffraction (EBSD) and transmission electron microscopy (TEM) before and after tensile tests to further clarify the plastic deformation mechanisms.

## Results

### Microstructures before tensile tests and quasi-static uniaxial tensile properties

**Figure 1:**
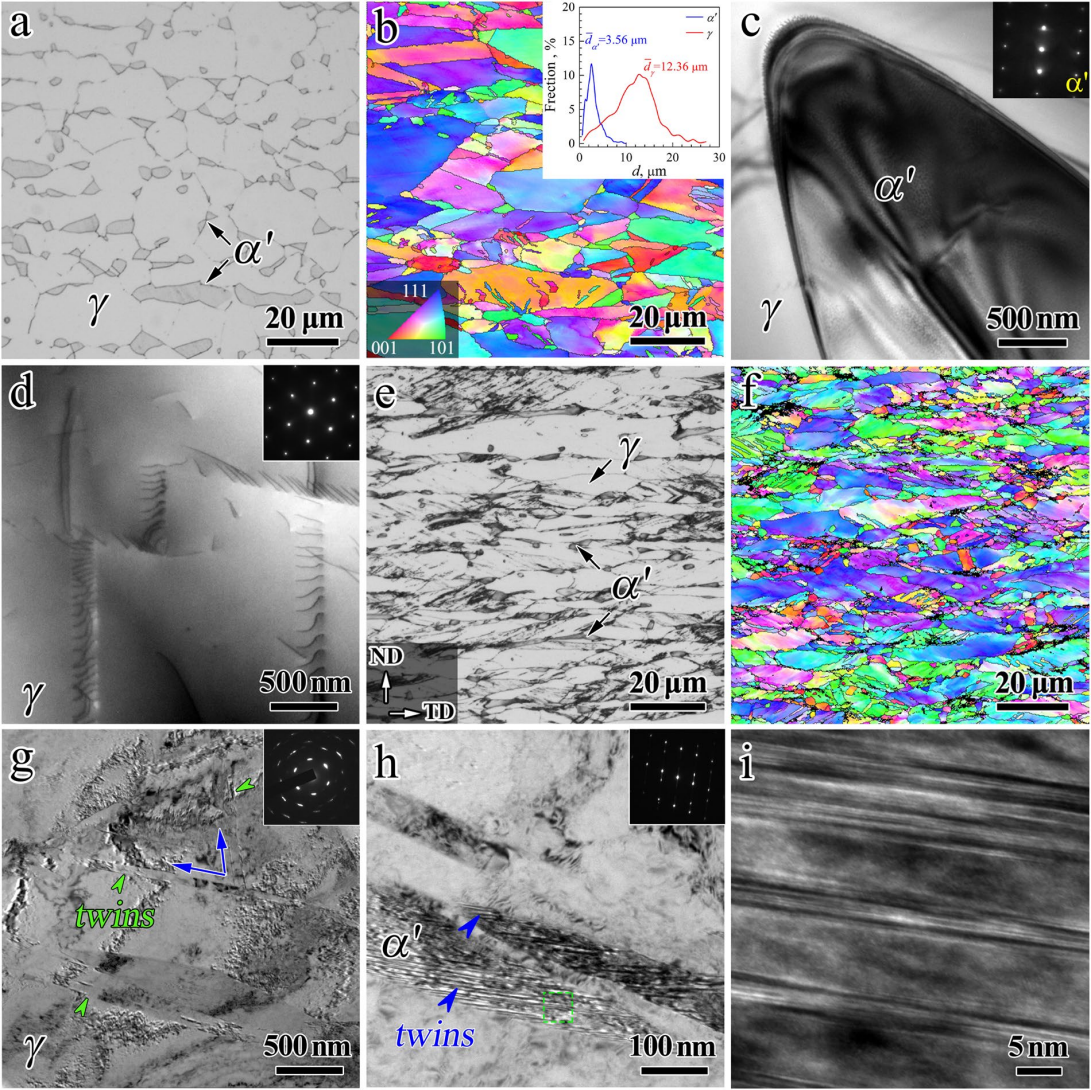
Microstructure characterizations for the solution treated and CR samples before tensile testing. (**a**,**b**) OM and EBSD (IPF) images for the solution treated sample, respectively; (**c**,**d**) TEM images for the solution treated sample; (**e**,**f**) OM and EBSD (IPF) images for the CR 50% sample, respectively; (**g**,**h**) TEM images for the CR 90% samples; (**i**) HREM image for the corresponding rectangle area in Fig. 1h.

The microstructure evolution during CR need be characterized first. Figure 1 shows the microstructures before the tensile tests for the solution treated sample and the CR samples with different thickness reductions. The optical microscope (OM) and EBSD (inverse pole figure, IPF) images for the solution treated sample are shown in Fig. 1a and b respectively. The TEM images for the solution treated sample are shown in Fig. 1c and d. As shown, the solution treated sample displays a dual-phase microstructure, composed of an austentite phase matrix (*γ* phase) with an average grain size of 12.4 μm and the second martensite phase (*α*′ phase) with an average grain size of 3.6 μm. It is observed that the *α*′ martensite grains are much inclined to precipitate at either the triple junctions or the grain boundaries of *γ* austenite grains. The area fraction is about 87% for the *γ* phase while is about 13% for *α*′ phase in the solution treated sample. As indicted in the TEM images for the solution treated sample, the grain interiors within both the *γ* and *α*′ grains are relatively clean due to the solution treatment at high temperature although a few dislocations can be seen.

Figure 1e and f display the OM and EBSD (IPF) images for the CR 50% sample. After CR with thickness reduction of 50%, the area fraction for the *α*′ phase is slightly increased to about 17% due to the martensite transformation during cold working. The plastic deformation is mainly carried by the heavily deformed structure in the soft *γ* grains and partially carried by the dislocation behaviors in the hard *α*′ grains during CR. As indicated, an inhomogeneous microstructure is observed for the CR 50% sample. A mixture of two areas, namely an area with large grain size of about 10 μm and an area with small grain size of about 1–3 μm or even sub-micron, is observed and this observation is consistent with that reported in previous research^27^. This indicates that the CR samples possess a hierarchical microstructure with both ultrafine grains (UFG) and coarse grains (CG) in both *γ* and *α*′ phases. After CR with thickness reduction of 90%, the microstructure can hardly be revealed by EBSD due to the even more heavily deformed structure. Thus, the detailed results for the severely deformed microstructure in the soft *γ* grains and the deformed microstructure in the hard *α*′ grains after CR with thickness reduction of 90% are displayed by TEM and high resolution electron microscope (HREM) images in Fig. 1g–i. Lamellae with thickness of about 50~100 nm are observed in the *γ* grains, and these lamellae are indentified to be deformation twins (DTs, marked by green arrows) with the exact twinning orientation relationship for fcc metals. As marked by blue arrows, multiple twins formed at two {111} planes are found in the grain interior of *γ* grains. High density of dislocation are also seen in the heavily deformed *γ* grains. Very high density of lamellar structures are observed in the hard *α*′ grains (as shown in Fig. 1h), and these structures are identified to be nanotwins (with thickness of a few nm) by the indexed selected area diffraction (SAD), which is confirmed by the HREM image of Fig. 1i. These microstructures (DTs and high density of dislocations) with nanoscale can result in the strengthening associated with CR.

**Figure 2:**
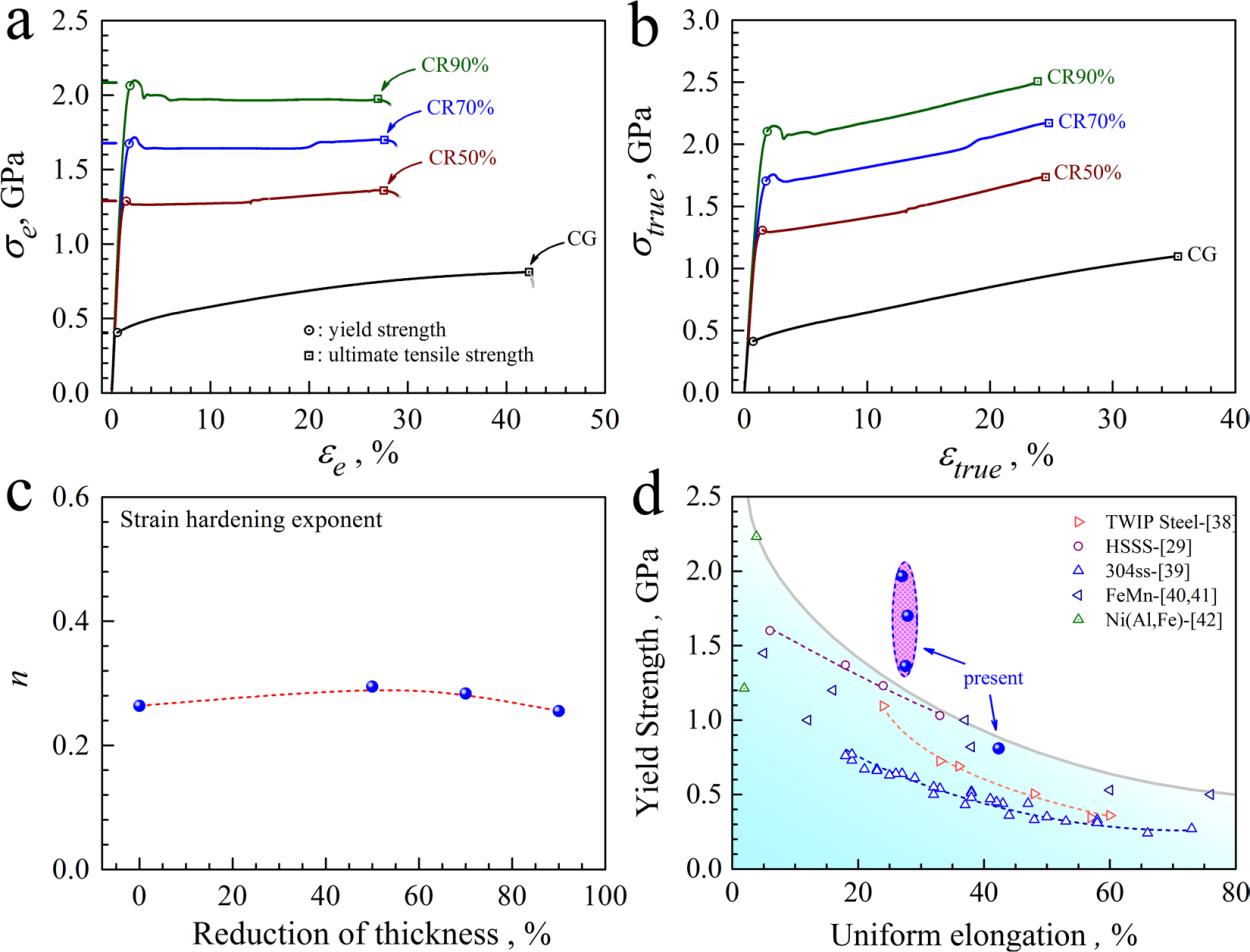
Tensile properties for the solution treated and CR samples. (**a**) Engineering stress-strain curves; (**b**) True stress-strain curves; (**c**) Strain hardening exponent versus the thickness reduction of CR; (**d**) Yield strength versus uniform elongation for the present data and other high-strength advanced steels.

The quasi-static uniaxial tensile properties for the solution treated sample and the CR samples are displayed in Fig. 2. Figure 2a and b show the engineering stress-strain curves and the true stress-strain curves, respectively. The yield points are marked by circles and the points for ultimate strength (UTS) are marked by squares in Fig. 2a and b. Then, the Holloman’s equation, $\sigma ={\sigma }_{0}+K{\varepsilon }^{n}$ (where *σ*_0_ is the yield stress, *K* is the strength coefficient, *n* is the strain hardening exponent), is used to fit the true stress-strain curves and the strain hardening exponent is plotted as a function of thickness reduction of CR in Fig. 2c. As indicated, the CG sample has a round continuous yielding and strain hardening behaviors, the yield strength is about 400 MPa and the uniform elongation is about 42%. While three-stages are observed before necking in the engineering stress-strain curves for the CR samples: a yield drop is followed by a stress plateau stage and a strain hardening stage. The strain duration for the stress plateau stage is observed to increase while the strain duration for the strain hardening stage is seen to decrease with the increasing thickness reduction for the CR samples. It is interesting to note that the yield strength is dramatically increased while the uniform elongation is still relatively reserved after CR. For example, the yield strength is elevated to about 2.1 GPa and the uniform elongation still remains about 26% after CR with thickness reduction of 90%. Compared to the CG sample, the strain hardening exponent is also observed to increase slightly after CR and be similar for all CR samples. The slope in the true stress-strain curves is observed to be similar for all samples, indicating that the CR process does not obviously reduce the strain hardening ability. As a summary, Fig. 2d plots yield strength versus uniform elongation curves for the present data and for other high-strength advanced steels^29,37–41^. The other conventional metals and alloys clearly show a banana curve for the trade-off between strength and ductility, while the present data exhibit ultrahigh strength and large tensile ductility with clear deviation from the other high strength metals and alloys. Specially, when the thickness reduction is increased from 50% to 90% for the CR samples, the yield strength is observed to be significantly elevated while the uniform elongation is found to be almost unchanged.

### Deformation mechanisms during tensile deformation for solution treated and CR samples

**Figure 3:**
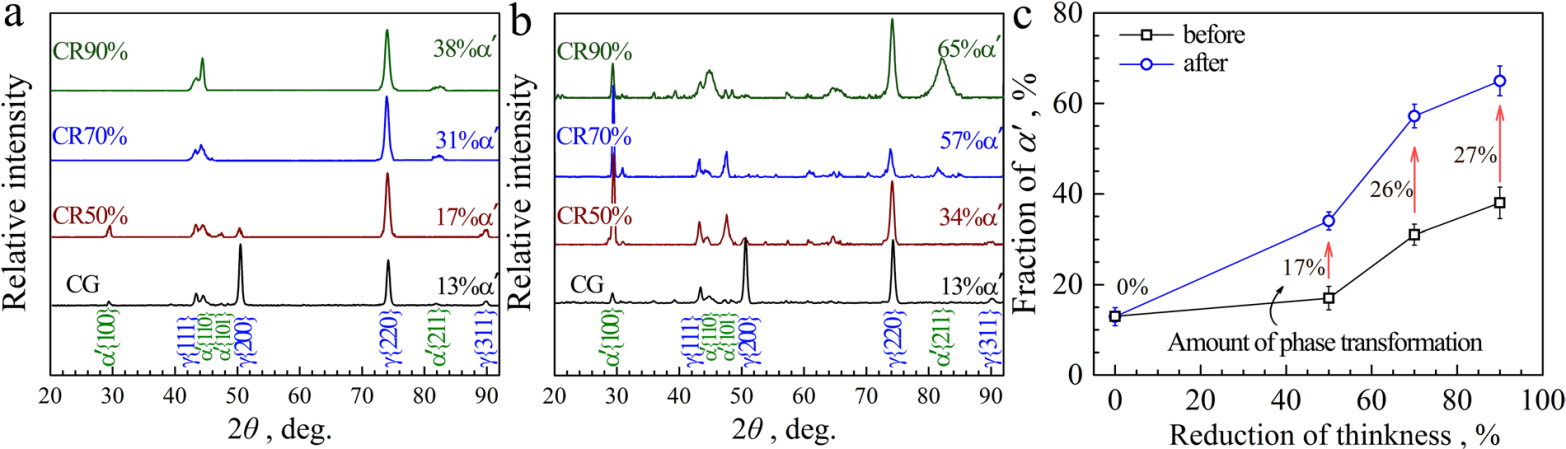
Martensite transformation during CR and subsequent tensile deformation. (**a**) The evolution of XRD spectra during CR; (**b**) The evolution of XRD spectra during subsequent tensile deformation; (**c**) The volume fractions of *α*′ phase as a function of CR thickness reduction for the solution treated and CR samples before and after tensile tests.

In order to reveal the deformation mechanisms for the solution treated and CR samples, the behaviors of martensite transformation during CR and subsequent tensile deformation need be quantitatively characterized. Thus, the XRD spectra in the solution treated sample and the CR samples with different thickness reductions are given in Fig. 3a, and the XRD spectra after tensile testing for all samples are shown in Fig. 3b. Based on these XRD spectra, the volume fraction of *α*′ martensite phase can be calculated using the same equations and methods as in our previous paper^42^. The volume fractions of *α*′ phase for the solution treated and CR samples before and after tensile tests are plotted as a function of thickness reduction of CR in Fig. 3c. The EBSD phase distributions for the solution treated and CR 50% samples before and after tensile testing are also shown in the Supplementary Information (Fig. S1). The volume fraction of *α*′ phase can also be counted from these EBSD phase images (Fig. S1), which is consistent with the observations from the XRD data (Fig. 3c).

As indicated, almost no phase transformation occurs during the tensile deformation for the solution treated sample. This observation indicates two aspects: (i) The soft *γ* CG, even the hard *α*′ grains, can deform plastically by deformation twins or dislocation behaviors without martensite transformation on one hand; (ii) It also shows that the austentite phase in this alloy is relatively stable and the flow stress for the CG sample is too low to reach the threshold stress and activate the martensite transformation on the other hand.

**Figure 4:**
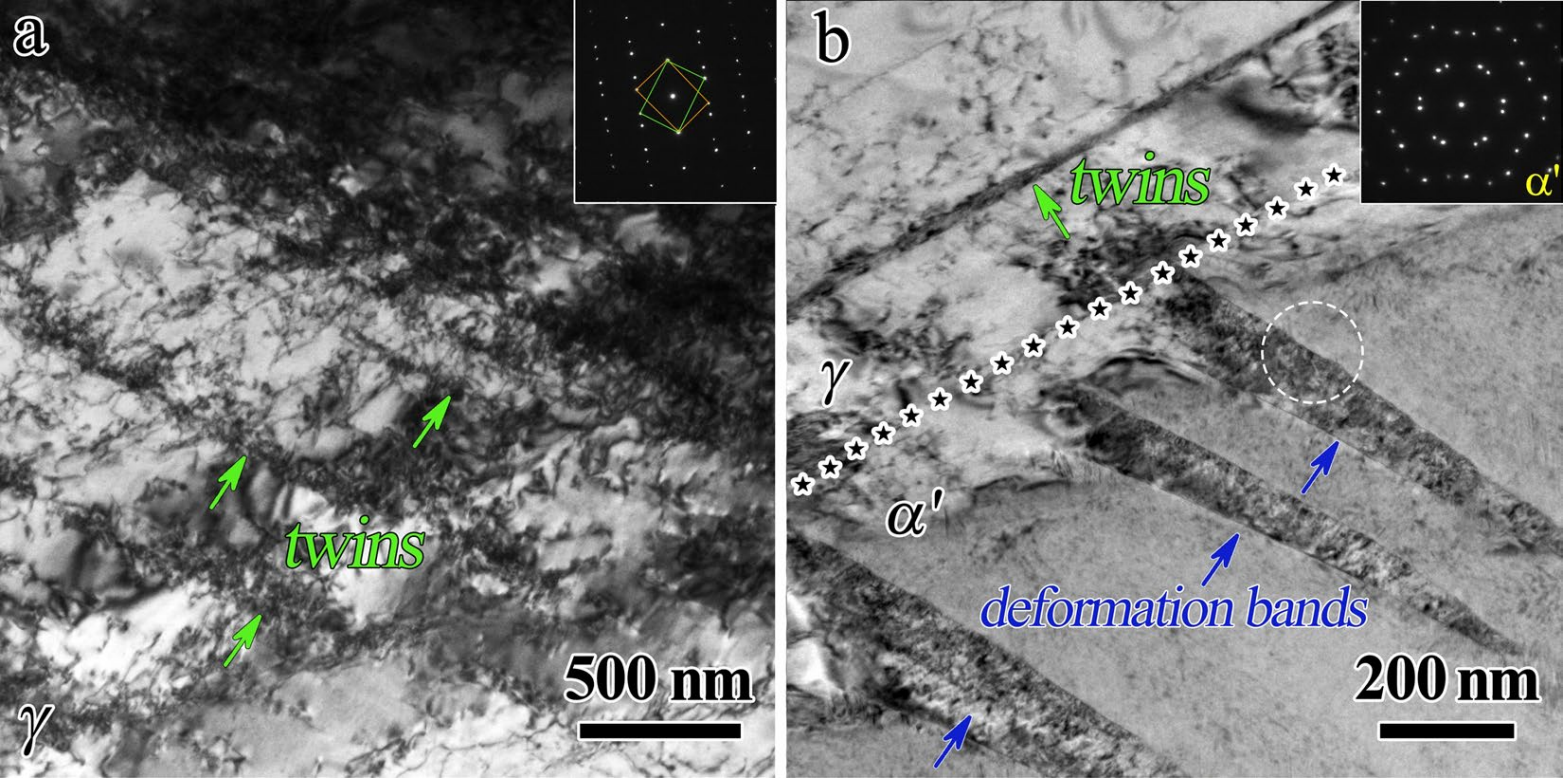
TEM observations for the solution treated sample after tensile deformation. (**a**) DTs and dislocations in the *γ* austentite grain; (**b**) Deformation bands and dislocations in the *α*′ martensite grain. The phase boundary between the *γ* austentite grain and the *α*′ martensite grain is marked by five-pointed stars.

In order to illustrate the deformation mechanisms for the solution treated sample, the uniaxial stress-relaxation results for the solution treated sample are displayed in Fig. S2, and the TEM images before and after tensile tests for the solution treated sample are shown in Fig. 4. The evolution of mobile dislocations can be examined through these repeated uniaxial stress-relaxation tests to reveal the strain hardening mechanism of the solution treated sample. Figure S2a displays the engineering stress versus engineering strain curve for the stress-relaxation test on the solution treated sample. The detailed data analysis for the repeated stress-relaxation tests can be found in our previous paper^7,43^. The calculated data are shown in Fig. S2b–d. Figure S2b shows physical activation volume as a function of engineering strain for the solution treated sample, Fig. S2c displays the exhaustion curves of mobile dislocation with respect to time at various preset strains for the solution treated sample, while Fig. S2d exhibits the retained density of mobile dislocation at the end of each relaxation against engineering strain. It is observed that the physical activation volume decreases while the mobile dislocation density increases with increasing engineering strain for the solution treated sample. As we know, the physical activation volume is proportional to the size of barrier for dislocations and the mean free path between barriers. Therefore, the mean free path generally decreases with increasing strain due to the increase of dislocation density and the multiplication of dislocations, thus resulting in reduction of physical activation volume. The increasing mobile dislocation density during the tensile loading for the solution treated sample indicates that dislocation behaviors in both phase should be a dominant deformation mechanism for the solution treated sample during tensile deformation, given that no obvious phase transformation is observed (as shown in Fig. 3c).

As shown in Fig. 1c and d, the dislocation density is low and the grain interior is relatively clean for both phases in the solution treated sample before tensile testing. While, high density of DTs (marked by green arrows, indicated by the indexed SAD in Fig. 4a) with a very small average twin boundary spacing (TBS) of several hundreds of nm are formed inside the soft *γ* grains after tensile deformation for the solution treated sample (Fig. 4a and b), and a few of deform bands with high density of dislocations are observed in the grain interior of *α*′ phase (Fig. 4b). The inset of Fig. 4b shows the indexed SAD for the area marked by white circle in the *α*′ phase, which clearly indicates the deformed structure by dislocations for bcc *α*′ phase. As observed in Fig. 4a, high density of dislocations near twin boundaries (TBs) are also observed in the grain interior of *γ* phase and TBs of DTs are no longer coherent due to the interactions between the dislocations and the TBs, which is consistent with the earlier results from the measurement of mobile dislocation density by stress relaxation tests. Previous research^11,12,44^ suggested that TBs of DTs are effective obstacles to the motion of dislocations and can accumulate the pile-up of dislocations near TBs, which could provide great resistance to the plastic deformation and strong strain hardening for sustaining uniform elongation.

As indicated, the volume fractions of *α*′ phase is slightly increased from 13% to 17% after CR with thickness reduction of 50%, and then increased to 31% after CR with thickness reduction of 70%, finally increased to 38% after CR with thickness reduction of 90%. These observations indicate that the martensite transformation also contributes to the strengthening during CR besides the microstructure refinement by UFG and DTs, and the increasing dislocation density inside grains. While, as indicated from results of both XRD data and EBSD images, significant amount phase transformation occurs during the tensile loading for CR samples, which is contrast to the CG sample (no phase transformation is observed during tensile deformation). For examples, the volume fractions of *α*′ phase is increased from 17% to 34% for CR 50% sample, from 31% to 57% for CR 70% sample, and from 38% to 65% for CR 90% sample during tensile deformation. The amounts of phase transformation during tensile testing are 17%, 26% and 27% for CR 50% sample, CR 70% sample and CR 90% sample, respectively (as shown in Fig. 3c). It is interesting to note that the amount of phase transformation increases with increasing thickness reduction of CR, which can result in strong strain hardening for sustaining excellent tensile ductility in the severely deformed alloy even the deformed grains by CR are insufficient to sustain the tensile deformation themselves. These observations indicate two things: (i) The plastic deformation ability by the soft *γ* CG or the hard *α*′ grains themselves almost exhausts during CR, and the plastic deformation during subsequent tensile loading need be accommodated by martensite tranformation; (ii) The flow stress for the CR samples is high enough to reach the threshold stress and activate the martensite transformation.

**Figure 5:**
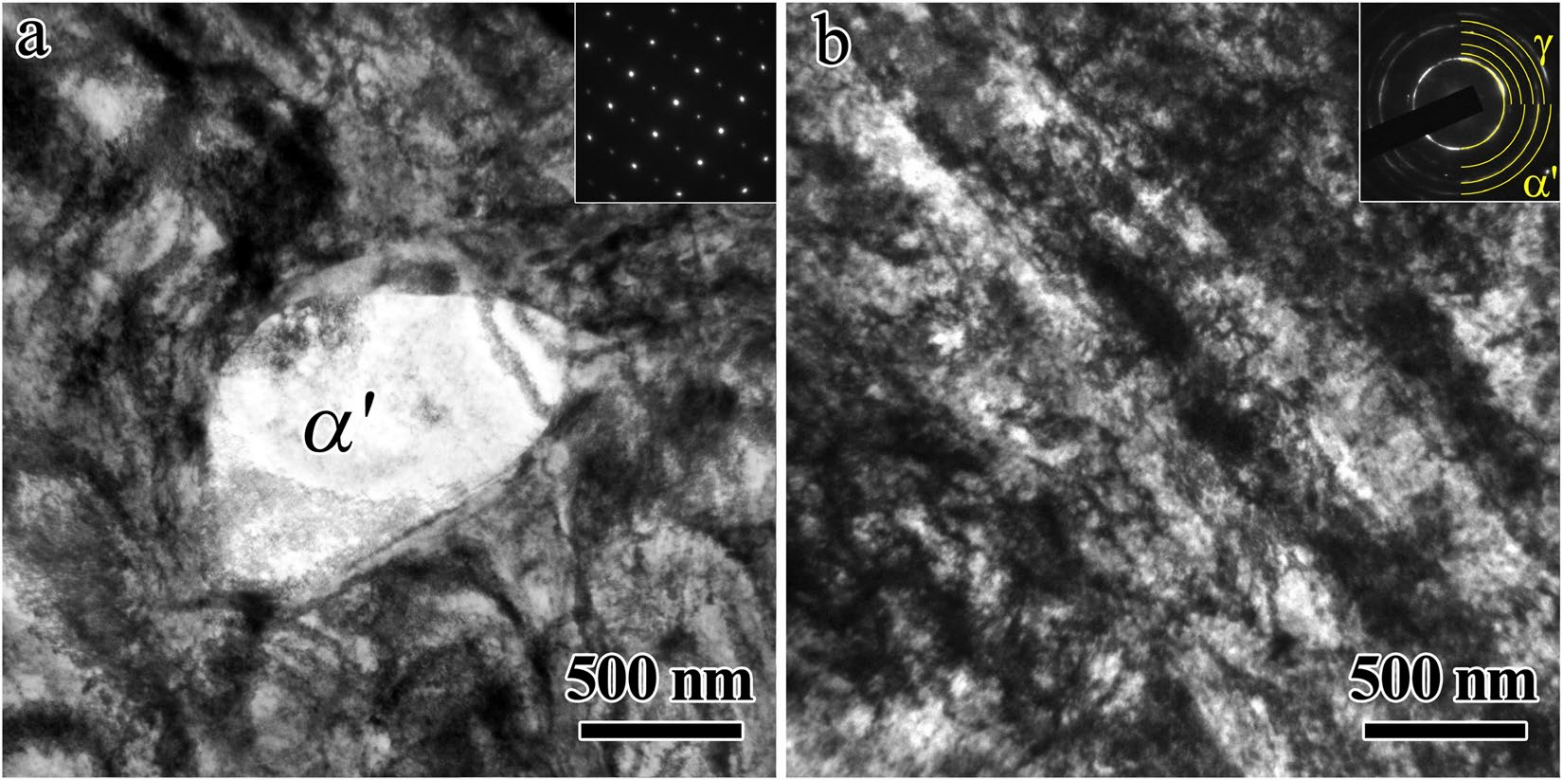
TEM observations for the CR 90% sample after tensile deformation. (**a**) Bright-field image showing accumulation of dislocations in the *γ* austentite grain around the *α*′ martensite nano-precipitate; (**b**) Bright-field image and the indexed SAD showing formation of nanograins and high density of dislocations in both phases.

The TEM images after tensile tests for the CR 90% sample are shown in Fig. 5. As indicated from Fig. 5a, high density of dislocations are accumulated in the *γ* grain around the *α*′ martensite nano-precipitate, which could result in strong strain hardening^5,13,14^. As indicated from Fig. 5b, nanograins are formed in both phases according to the indexed SAD, even higher density of dislocations are observed within both *γ* grains and *α*′ grains after tensile testing when compared to those before the tensile deformation for the CR 90% sample. Thus, the strong hardening for the CR samples during the tensile deformation could be attributed to two aspects: (i) The obvious martensite transformation can contribute to the strain hardening and accommodate the plastic strain since the martensite phase is harder than the austenite phase. This stress-induced transformation can provide the transformation strain itself to prevent early void formation at the phase interfaces on one hand, and can strengthen the strain concentration region and thus help to maintain large homogeneous tensile deformation by preventing early necking formation on the other hand^19,45^. (ii) Followed CR, the continuous interactions between dislocations and TBs during the tensile deformation could involve the formation of nanograins and immobile dislocation locks for strengthening and hardening^44^, and the accumulation of dislocations around the *α*′ martensite nano-precipitates could also result in strengthening and hardening^5,13,14^.

## Discussions

As indicated in our previous paper^30^, the high strain hardening and the excellent ductility in the HSSS with dual-phase microstructure can be attributed to the high back stresses that arise from plastic deformation incompatibility between the two phases with different mechanical properties. The back stress hardening has been found to also play an important role in the heterogeneous lamella structure Ti with both UFG and CG lamellae^10^. Thus, the back stress hardening should also play an important role in the current alloy with dual-phase microstructure. The back stress hardening might contribute more to the strain hardening and the ductility in the CR samples than the solution treated sample due to the fact that the initial microstructure for the CR samples is hierarchical with both UFG and CG in both phases and this heterogeneous microstructure is dynamically reinforced due to the significant martensite transformation during tensile deformation for the CR samples.

Figure S3a,b shows Vickers micro-hardness 2D contours for the CG sample and the CR 50% sample before and after tensile tests. Then the corresponding micro-hardness distributions are plotted in Fig. S3c,d. As indicated, the average hardness is increased from 420 to 462 Hv for the solution treated sample, and from 550 to 663 Hv for the CR 50% sample after tensile deformation. It is interesting to note that the hardness increment is larger for the CR 50% sample than for the solution treated sample, which could be due to the martensite tranformation for the CR 50% sample. Another interesting thing to note is that a wide distribution of micro-hardness is observed for both the solution treated sample and the CR samples, which indicates that the microstructures are highly heterogeneous with different mechanical properties at different areas for both the solution treated sample and the CR samples. These heterogeneous microstructures with different yield strengths/hardness at different areas should result in strong stress/strain partitioning at different areas. For the solution treated sample, this strain partitioning generally happens between two phases (i.e., the softer *γ* grains accommodate more plastic strain and achieve more strain hardening than the harder *α*′ grains during tensile loading). This strain partitioning can be measured for both phases by the aspect ratio changes before and after tensile tests in the solution treated sample since no phase transformation occurs. It is observed that the softer *γ* grains are more severely elongated along the tensile direction (horizontal direction) than the harder *α*′ grains, as indicated in Fig. S1a and b. These results can qualitatively indicate that the strong strain partitioning indeed happens between two phase with different mechanical properties. Thus, this plastic incompatibility due to the load transfer and the strain partitioning between two phases is the main origin for the back stress hardening in the solution-treated sample.

In order to understand the deformation mechanism of the stress plateau stage observed for the CR samples, additional *in-situ* digital image correlation (DIC) experiment along with tensile testing was also conducted for the CR 70% sample. The evolution of strain contours for the gauge section along with tensile strain is shown in Fig. S4. It can be clearly seen that the stress plateau stage for the CR 70% sample is due to the nucleation and propagation of the deformation band (Lüders band). This discontinuous yielding followed by large Lüders strain is similar to the results observed for a deformed and partitioned high strength steels in the recent work^46^, and this phenomenon can help to sustain large ductility.

**Figure 6:**
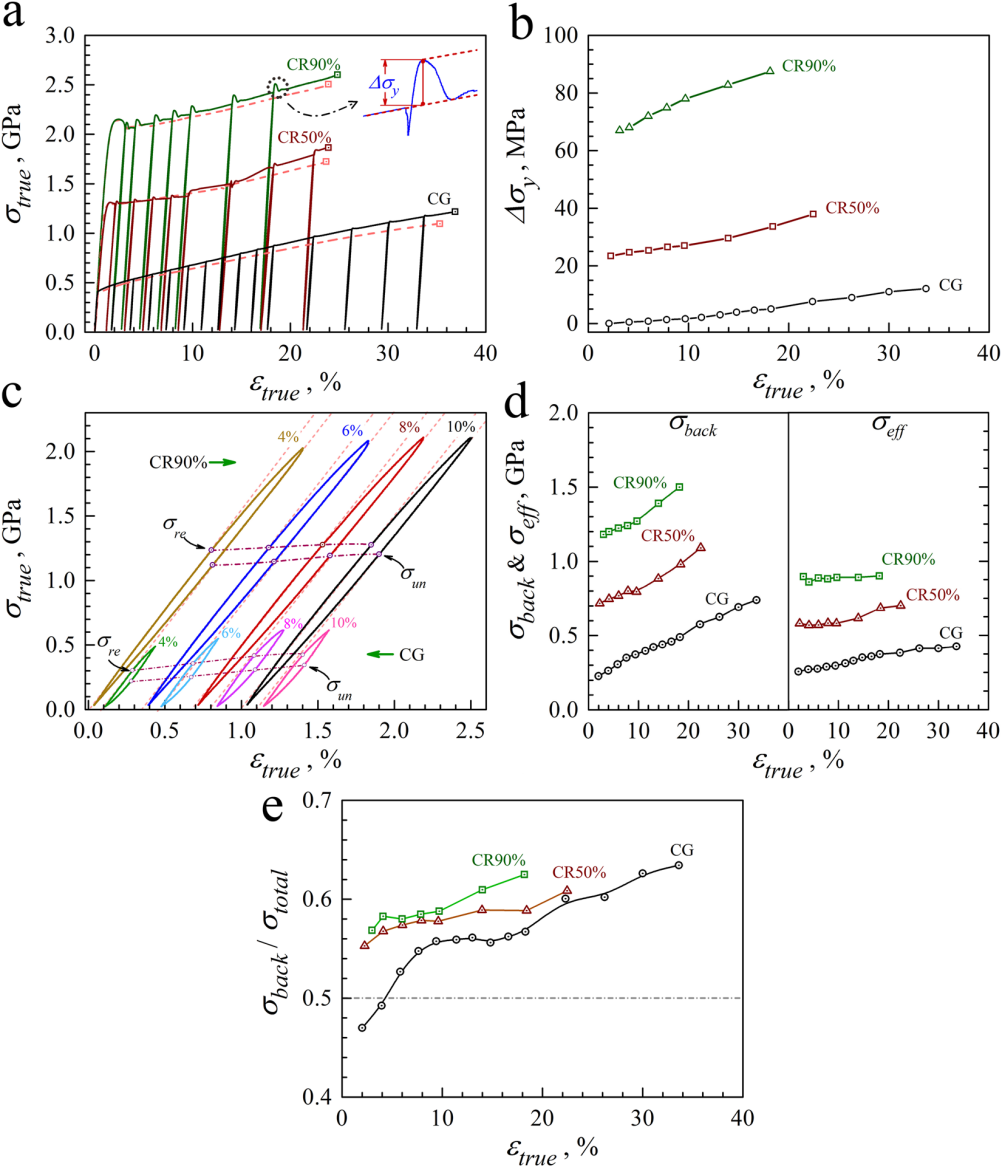
Bauschinger effect and back stress hardening for the solution treated and CR samples. (**a**) The true stress-strain curves for LUR tests; (**b**) ${\rm{\Delta }}{\sigma }_{y}$ as a function of true tensile strain due to unloading yield effect; (**c**) The close-up views of typical hysteresis loops; (**d**) The evolutions of ${\sigma }_{back}$ and ${\sigma }_{eff}$ along with tensile strain; (**e**) The evolution of ${\sigma }_{back}/{\sigma }_{total}$ along with tensile strain.

The true stress-strain curves for LUR tests on the solution treated sample, the CR 50% sample and the CR 90% sample are shown in Fig. 6a, and the inset displays the close-up view for the yield drop phenomenon in the reloading curve of the CR samples. According to our previous paper^30^, this unloading yield effect could be understood as follows: once unloaded, the hard *α*′ grains become elastic. Upon reloading, the hard *α*′ grains stays elastic while the soft *γ* grains begins to deform plastically. Upon reloading, the yield peak appears due to the load transfer between two phases. Then, rapid relaxation of elastic stresses and strains at the phase interfaces causes the stress drop once the hard *α*′ grains yields upon reloading. As shown in Fig. 6b, the unloading yield effect is much obvious in the CR samples than in the solution treated sample due to the facts that the volume fraction of hard *α*′ phase is higher and is significantly increased during tensile deformation for the CR samples.

The close-up views of typical hysteresis loops for the solution treated sample and the CR samples are shown in Fig. 6c, and the back stress can be estimated by the average of the unloading yield stress and the reloading yield stress (${\sigma }_{back}=({\sigma }_{u}+{\sigma }_{r})/2$), which was proposed in our previous paper^47^. The effective stress ${\sigma }_{eff}$, which contributes to the forest dislocation hardening, can be calculated by detracting the back stress from the total flow stress. Then the evolutions of both the back stress and the effective stress along with tensile strain can be obtained from the unloading-reloading curves at varying tensile strains, and are shown in Fig. 6d. Moreover, the evolution of ${\sigma }_{back}/{\sigma }_{total}$ along with tensile strain is shown in Fig. 6e for the solution treated sample and the CR samples. The magnitudes of ${\sigma }_{back}/{\sigma }_{total}$ for all samples increase with increasing strain, indicating the high back stress hardening for all samples. ${\sigma }_{back}/{\sigma }_{total}$ can represent the contribution of back stress hardening to the overall strain hardening, thus the contribution of back stress hardening is observed to be much larger for the CR samples than for the solution treated sample. The back stress hardening can be only originated from the long internal stress between the soft *γ* phase and the hard *α*′ phase for the solution treated sample. While, the back stress hardening is accommodated by the pile-up and accumulation of geometrically necessary dislocations at the phase interfaces or at the boundaries between UFG and CG for the CR samples, which is caused by the long-range internal stress among the *γ* CG, the *γ* UFG and the hard *α*′ grains. Thus, stronger back stress hardening in the CR samples could be attributed to the much more complex interplay among the *γ* CG, the *γ* UFG and the hard *α*′ grains and the dynamically reinforced heterogeneous microstructure in the CR samples due to the significant phase transformation.

In summary, the plastic deformation mechanisms of the solution treated sample and the CR samples in a Fe-24.8%Ni-6.0%Al-0.38%C alloy have been investigated in the present work, and the main findings are summarized as follows. The yield strength is dramatically elevated after CR without significant reduction of the uniform tensile ductility. The present results exhibit ultrahigh strength and large tensile ductility with clear deviation from the trade-off banana curve for the other high strength metal and alloys. The austentite in this alloy is relatively stable and no obvious phase transformation occurs during the tensile loading of the solution treated sample. Deformation twins and dislocation behaviors in the CG are the main plastic deformation carriers during the tensile deformation for the solution treated sample. Obvious phase transformation was observed during the tensile loading for the CR samples, and the amount of phase transformation was found to increase with increasing thickness reduction of CR, resulting in excellent tensile ductility in the severely deformed alloy. On the one hand, the deformation ability of the CG exhausts during CR and the deformed CG by CR are insufficient to sustain the tensile deformation themselves. On the other hand, the tensile flow stress of the CR samples is high enough to activate the martensite transformation. The tensile ductility in this alloy with dual-phase microstructure can be attributed to the strong back stress hardening due to the load transfer and the strain partitioning between the two phases, and the back stress hardening was observed to play a more important role in the CR samples than in the solution treated sample due to the much more complex interplay among the *γ* CG, the *γ* UFG and the hard *α*′ grains, and the dynamically reinforced heterogeneous microstructure by the phase transformation. The present results could provide better understanding for the deformation mechanisms of the Fe-Ni-Al-C alloy and could provide strategies to achieve both ultrahigh strength and excellent tensile ductility in metals and alloys.

## Materials and Experimental procedures

### Materials

The Fe-24.8Ni-6.0Al-0.38 C (in wt.%) alloy was first melted in an induction furnace under the protection of Ar atmosphere, and then cast into ingots with dimensions of 750 × 120 × 120 mm^3^. The ingots were then hot forged between 1423 and 1223 K into slabs with a thickness of 6 mm. Next, the slabs were solution treated at 1373 K for 24 h in Ar atmosphere and were then quenched in water. After solution treatment, the slabs were cold rolled into sheets with different thickness reductions (50%, 70%, and 90%).

### Microstructure characterizations

OM, EBSD and TEM were used to study the microstructures before and after the tensile tests. The sample surfaces for OM and EBSD were first grinded to 3000 grit with sandpapers, and then polished with a 0.05 μm SiO_2_ aqueous suspension, followed by electro-polishing in a solution of 5% HClO_4_ and 95% alcohol at 37 V and 253 K (−20 °C). For TEM observations, thin disks with thickness of about 200 μm were prepared, and then mechanically polished to about 40 μm, followed by a twin-jet polishing using a solution of 5% perchloric acid and 95% ethanol at 25 V and 253 K (−20 °C). X-ray diffraction (XRD) measurements were performed on polished samples to obtain the phase transformation information during CR and tensile tests using a Philips Xpert X-ray diffractometer with Cu Kα radiation. The phase volume fractions were estimated from the peak integrated intensities I_hkl_ after background subtraction.

### Mechanical testing

The specimens for quasi-static uniaxial tensile tests and LUR tests have a dog-bone plate shape and a gauge section of 10 × 4 × 0.5 mm^3^. The tensile direction is parallel to the rolling direction. These tensile tests and LUR tests were conducted at a strain rate of 5 × 10^−4^/s and at room temperature under displacement control using an Instron 5565 testing machine. In order to obtain the evolution of mobile dislocation density during the tensile tests, uniaxial tensile stress-relaxation tests were also performed under strain-control mode at room temperature with a series of preset strains. Upon reaching a designated relaxation strain, the strain was maintained constant while the stress was recorded as a function of time. After the first relaxation over an interval of 90 s, the specimen was reloaded by a strain increment of 0.6% at a strain rate of 5 × 10^−4^/s for next relaxation. Four stress relaxations were conducted for each designated strain. During the tensile tests, LUR tests and tensile stress-relaxation tests, an exensometer was used to accurately measure and control the strain. The distributions (2D contours) of Vickers micro-hardness before and after tensile tests were also obtained on the polished sample surfaces using a Vickers diamond indenter under a load of 5 g for 15 s dwell time. The area for micro-hardness measurement is 140 × 210 μm^2^, and the distance for each indentation is about 5 μm. Strain contours were measured using DIC during tensile tests. A commercial software, ARAMIS^®^, was utilized to analyze the DIC data. Initial high-contrast stochastic spot patterns on the sample surface were created. The evolution of the spot patterns was recorded using two 1.2 MPx digital CCD cameras at a rate of 1 frame per second. The facet size for the strain calculation using DIC method was 50 μm. The other details for the DIC method can be found in our recent paper^48^.

### Availability of materials and data

The authors declare no restrictions on the availability of materials and data.

## Electronic supplementary material

Supporting information
