# Supplementary material for: Plastic deformation mechanisms in a severely deformed Fe-Ni-Al-C alloy with superior tensile properties
Source: Sci Rep. 2017 Nov 15;7:15619. doi: 10.1038/s41598-017-15905-5 (PMC5688163; doi:10.1038/s41598-017-15905-5)
Supplement: Supplementary file 1 — Supporting information [file 41598_2017_15905_MOESM1_ESM.pdf]

Plastic deformation mechanisms in a severely deformed Fe-Ni-Al-C alloy with superior tensile properties

Yan Ma[1,2], Muxin Yang[1], Ping Jiang[1], Fuping Yuan[1,2*] & Xiaolei Wu[1,2]

[1] State Key Laboratory of Nonlinear Mechanics, Institute of Mechanics, Chinese Academy of Sciences, Beijing 100190, China

[2]School of Engineering Science, University of Chinese Academy of Sciences, Beijing 100190, People's Republic of China

Supplementary Information

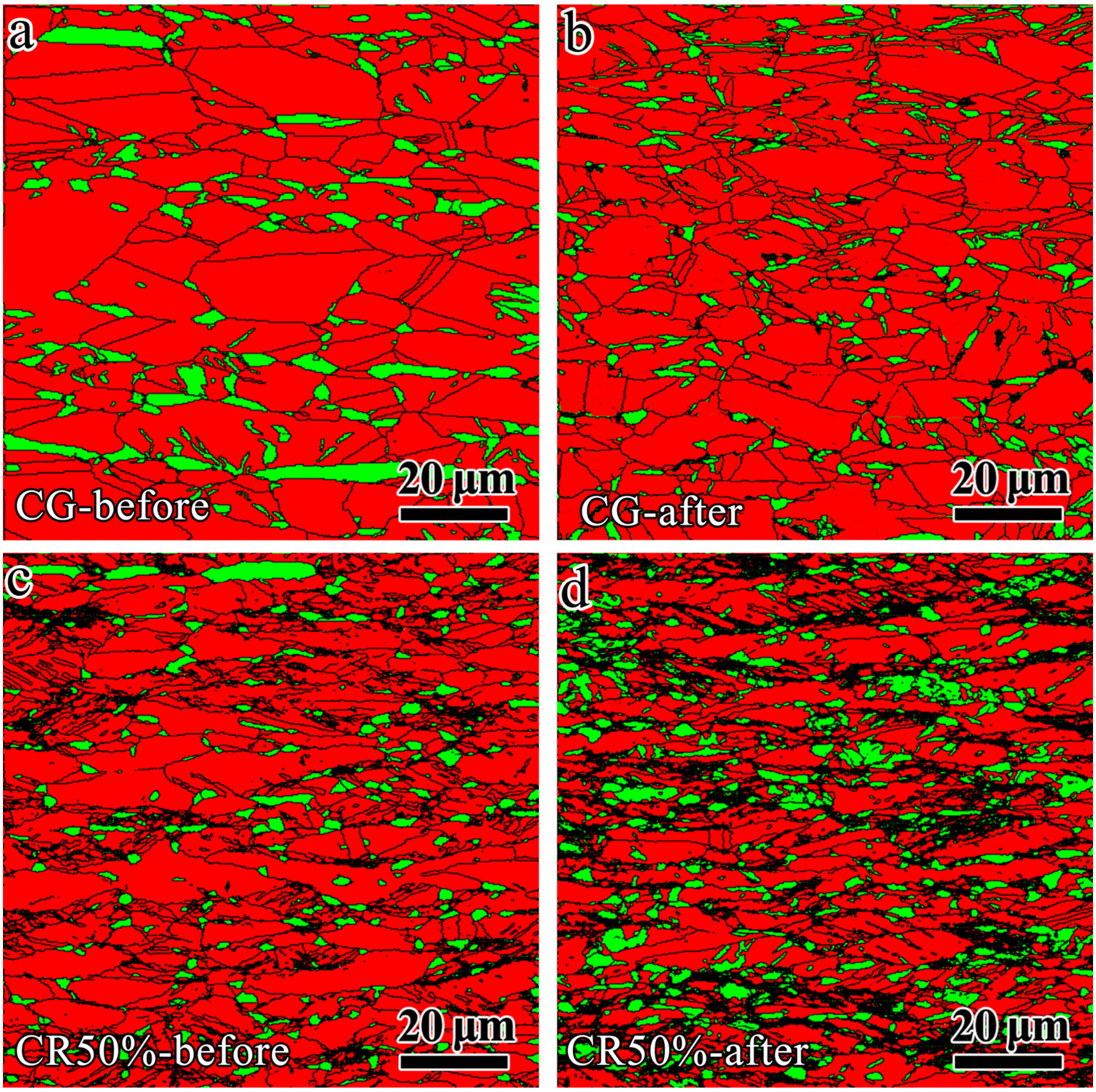

Figure S1. (a) (b) EBSD phase distributions for the solution treated sample before and after tensile deformation, respectively; (c) (d) EBSD phase distributions for the CR 50% sample before and after tensile deformation, respectively.

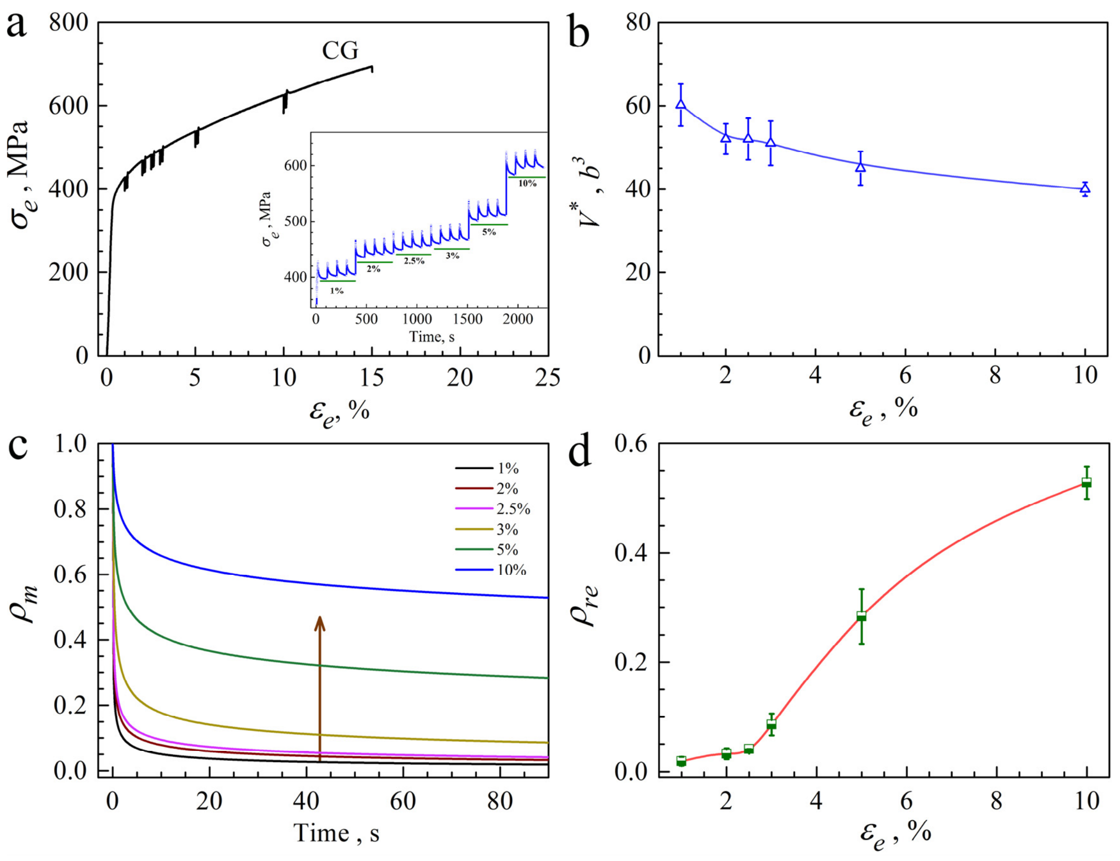

Figure S2. (a) Engineering stress-strain curve for the stress-relaxation test on the solution treated sample; (b) Physical activation volume as a function of engineering strain for the solution treated sample; (c) Exhaustion curves of mobile dislocation with respect to time at various preset strains for the solution treated sample; (d) Retained density of mobile dislocation at the end of each relaxation against engineering strain for the solution treated sample.

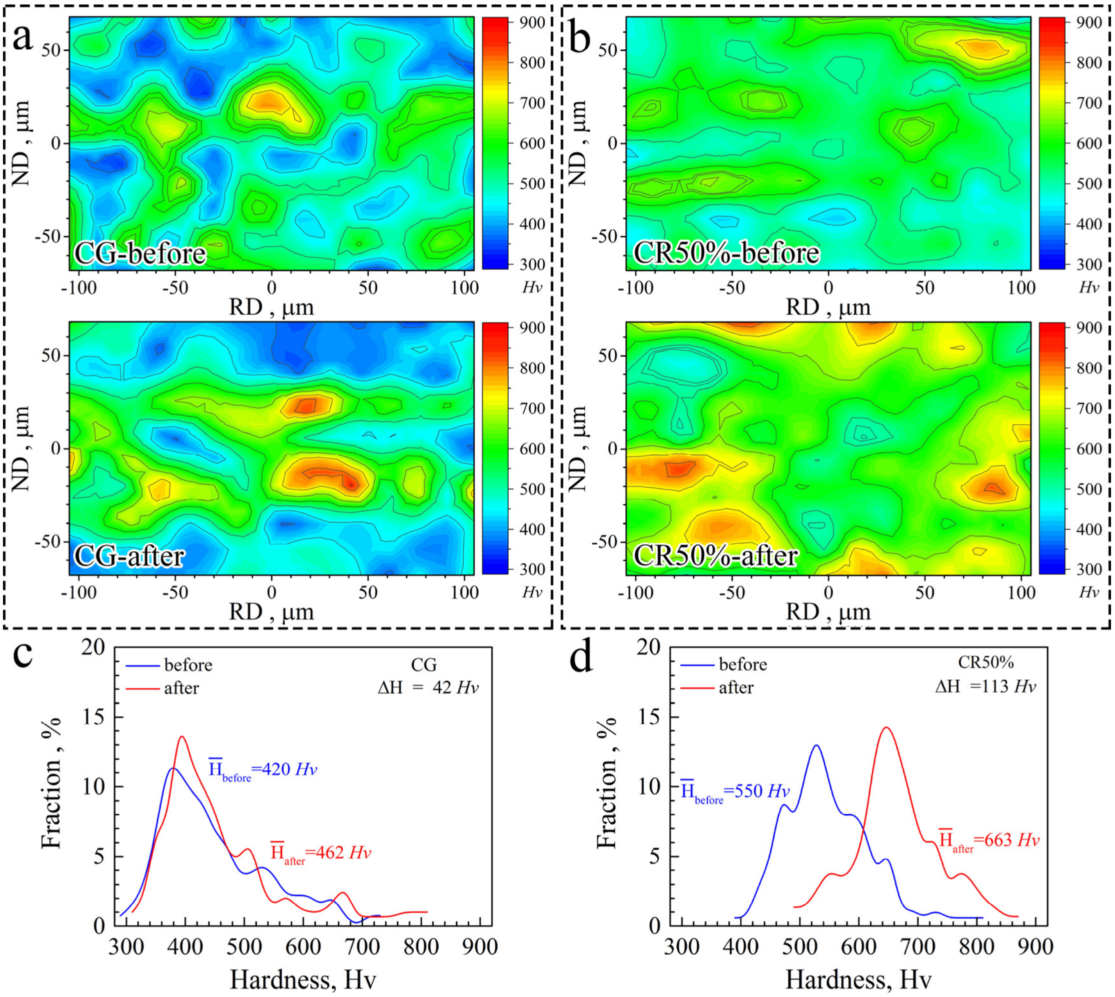

Figure S3. Vickers micro-hardness 2D contours before and after tensile deformation for (a) the solution treated sample; (b) the CR 50% sample. The corresponding micro-hardness distributions before and after tensile deformation for (c) the solution treated sample; (d) the CR 50% sample.

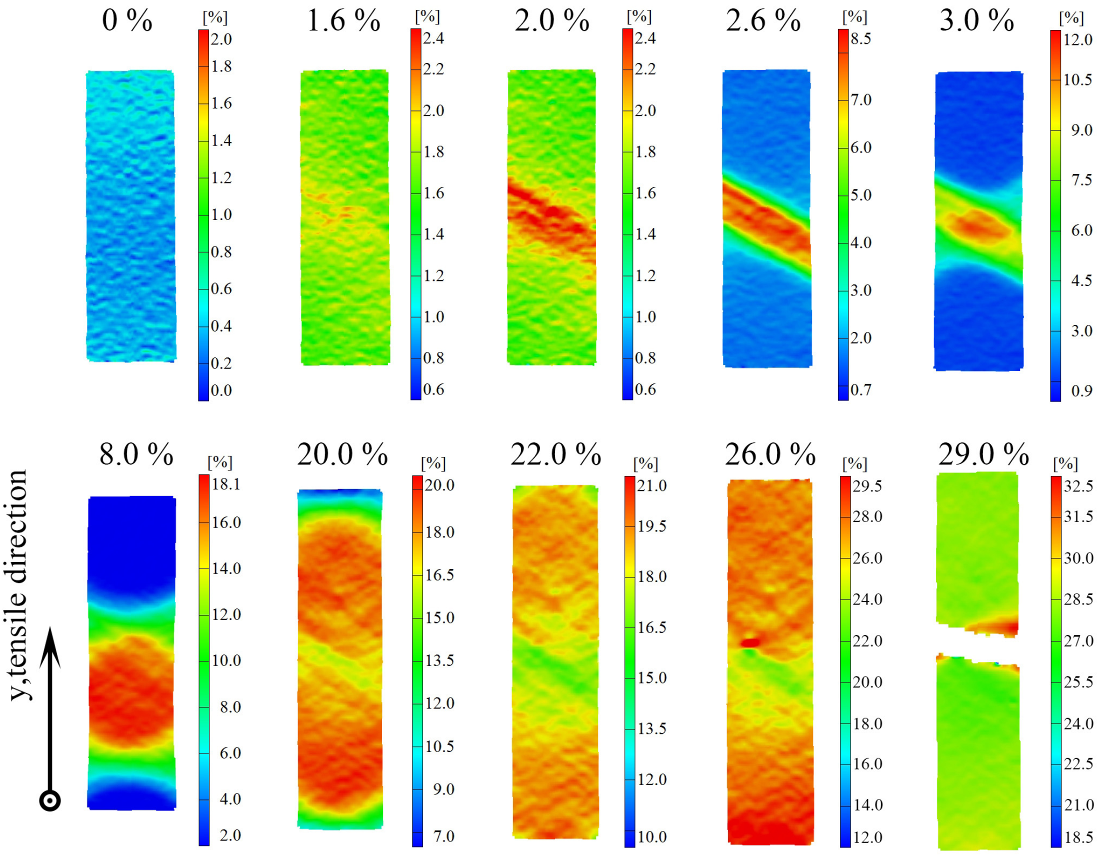

Figure S4. The evolution of strain contours for the gauge section along with tensile strain for the CR 70% sample.
